# Cloning and expression of the EsxA gene and the growth-promoting effects of the encoded protein on rice seedlings

**DOI:** 10.1186/s13568-021-01234-4

**Published:** 2021-05-25

**Authors:** Wen-qing Yu, Xin Wang, Yi-cong Tang, Feng-chao Yan, Wen-zhi Liu, Gui-ping Zheng, Dong-mei Yin

**Affiliations:** 1 College of Life Sciences, Shangrao Normal University, Shangrao Agricultural Technology Innovation Institute, Shanrao, Jiangxi 334001 China; 2 Heilongjiang Academy of Agricultural Reclamation Sciences, Haerbin, 150038 China; 3 Heilongjiang Bayi Agricultural University, Daqing Heilongjiang, 163319 China; 4 China Quality Certification Centre, Beijing, 100070 China

**Keywords:** EsxA, *Paenibacillus terrae*, Gene expression, Elicitor, Growth promotion, Rice

## Abstract

An EsxA-encoding gene (*esxA*) was previously identified in the genome of the plant growth-promoting rhizobacterium *Paenibacillus terrae* strain NK3-4. The *esxA* was cloned and expressed in *Pichia pastoris*, after which the effects of the EsxA protein on rice seedling growth were analyzed to determine whether EsxA contributes to the plant growth-promoting activity of strain NK3-4. The *esxA* was successfully cloned from the NK3-4 genome and ligated to the eukaryotic expression vector pPICZαA. The resulting pPICZαA-*esxA* recombinant plasmid was transinfected into yeast cells, and *esxA* expression in the yeast cells was confirmed. The treatment of seed- buds with the EsxA protein increased the root length by 1.35-times, but decreased the bud length. Additionally, in rice seedlings treated with EsxA, the root and shoot lengths increased by 2.6- and 1.7-times, respectively. These findings imply that EsxA is important for the promotion of rice plant growth by *P. terrae* strain NK3-4. Furthermore, the construction of the *esxA* expression vector and the engineered strain may be useful for future investigations of the mechanism underlying the plant growth-promoting effects of EsxA, with implications for the application of EsxA for regulating plant growth.

## Introduction

Early secreted antigenic target of 6 kDa (ESAT-6), which is a member of the WXG super family, is encoded by the EsxA gene (*esxA*). This protein, which comprises approximately 100 amino acids, belongs to the Type VII secretion system. Additionally, it forms a dimer structure under natural conditions. The EsxA protein was first identified in the animal pathogen *Mycobacterium tuberculosis* (Pollock and Andersen 1997), and was revealed to be important for pathogenicity (Berthet et al. 1998; Ulrichs et al. 1998). The protein was subsequently detected in other animal pathogens (Schulthess et al. 2012; Ma et al. 2015), and has been studied as a virulence factor for bacterial pathogens. However, EsxA is not a simple virulence factor, in addition to being important for the pathogenicity of animal pathogens, it can also induce an immune response in animals (Yi et al. 2018). For example, *Staphylococcus aureus* EsxA can promote antibody production in patients infected with this bacterium (Zhou et al. 2013).

In an earlier study, we identified an *esxA* in the genome of the plant growth-promoting rhizobacterium *Paenibacillus terrae* strain NK3-4 (Yu et al. 2019a). A phylogenetic analysis based on the EsxA amino acid sequences of *Paenibacillus* strains revealed that *esxA*s are widely distributed in plant growth-promoting *Paenibacillus* strains (Yu 2019). However, there are no reports describing the functions of EsxA in *Paenibacillus* strains or in other non-pathogenic bacteria, and it is unclear whether EsxA contributes to the plant growth-promoting effects of specific rhizobacteria. In this study, an *esxA* will be cloned from *P. terrae* NK3-4 genome and heterologous expressed in *Pichia pastoris* cells, and then applied to rice to investigate the role it plays on growth. The results of this study may be useful for clarifying the mechanism by which *Paenibacillus* strains, including NK3-4, promote plant growth.

## Materials and methods

### Materials

The plant growth-promoting rhizobacterium *P. terrae* strain NK3-4 used in this study was isolated and identified in one of our earlier studies (Yu et al. 2014), and was maintained in our laboratory. *Pichia pastoris* KM71H and the pPICZαA vector were provided by the Institute of Plant Protection, Chinese Academy of Agricultural Sciences. The pPICZαA vector was supplied by Institute of Plant Protection, Chinese Academy of Agricultural Sciences, now stored in our library. The size of the pPICZαA vector is 3593 bp, resistance marker is zeocin, promoter is AOX1, the restriction sits of *Eco*RI and *Xba*I is included. Tobacco (*Nicotiana tabacum*), tomato (*Solanum lycopersicum*), radish (*Scrophularia ningpoensis*), and pakchoi (*Brassica rapa* L. ssp. *chinensis*) plants were grown in pots for use.

### Cloning, expression, and purification of esxA

#### Extraction of strain NK3-4 genomic DNA

Genomic DNA was extracted from strain NK3-4 using the Bacterial Genomic DNA Extraction kit (Beijing Solarbio Technology Co., Ltd., Beijing, China). The extracted DNA was stained with GoldView II Nuclear Staining Dyes (Beijing Solaibao Technology Co., Ltd, Beijing, China) and analyzed by 1 % agarose gel electrophoresis (AGE) to check it was high molecular weight and not sheared. Additionally, the OD_260_/OD_280_ ratio was examed using ultra-micro ultraviolet spectrophotometer (NanoDrop one, Thermo Fisher Scientific, Waltham, Massachusetts, USA), The examination showed that the OD_260_/OD_280_ ratio is 1.8–2.0, indicating that the genomic DNA was free of proteins and RNA, was appropriate for the subsequent gene cloning.

#### Cloning of the esxA

**Fig. 1:**
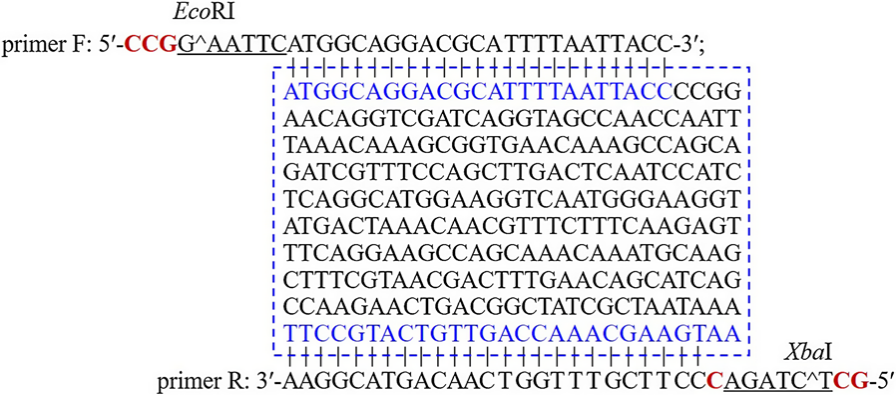
Informations of esxA and primers Full length of *esxA* (in squre frame), primers with recognized bases and restriction sites of *Eco*RI and *Xba*I as well as protective bases (bold in red letters) are shown

Primer design: the following primers were designed according to the *esxA* sequence (Fig. 1), the GenBank accesion number of *esx*A in GenBank of NCBI is SAMN16617042) in the NK3-4 draft genome database (undisclosed). The *Eco*RI (GAATTC)/*Xba*I (TCTAGA) recognized bases (restriction sites is shown in the primer sequences) as well as protective bases (as bold in red letters the primer sequences) were added tothe 5′-ends of the pair of primers. The addiation of protective bases was to prevent the nucleases (*Eco*RI and *Xba*I) from cleavaging the DNA at wrong sites (Fig. 1).

PCR amplification of the coding gene of EsxA: The PCR amplification was completed in a 50-µL solution comprising 25 µL 2 × HiFiMix I, 20 µL ddH_2_O, 1 µL DNA template, and 2 µL each primer (10 µM). The PCR program was as follows: 95 °C for 5 min; 35 cycles of 94 °C for 30 s, 56 °C for 30 s, and 72 °C for 1 min; 72 °C for 10 min. The PCR product was maintained at 4 °C until it was analyzed and purified. The DNA sequence of *esxA* as show in Fig. 1 (in squre frame). The predicted amplicon size is 292 bp.

#### Purification of the PCR product

The PCR product was stained with the GoldView II Nuclear Staining Dyes and analyzed by 1 % gel electrophoresis, then purified using the QIAquick Gel Extraction Kit (Qiagen, Frankfurt, Germany). The quality and concentration of the recovered DNA were checked, after which the DNA was stored at − 20 °C.

#### Amplification of the pPICZαA vector

The pPICZαA vector was transformed into competent *Escherichia coli* Trans1-T1 cells (Beijing TransGen Biotech. Co., Ltd, Beijing, China). Positive transformants screened on solid LA medium containing bleomycin (500 µg/mL) were used to inoculate liquid LB medium supplemented with bleomycin (500 µg/mL). The pPICZαA vector was purified from the *E. coli* cells using Easy Pure Plasmid MinPrep Kit (Beijing TransGen Biotech. Co., Ltd, Beijing, China) according the operation manual, after which the recovered vector quality was checked through AGE, and the pPICZαA (1 µL) was add on the ultra-micro ultraviolet spectrophotometer to determin the concentration. The purified amplified pPICZαA was stored at − 20 °C.

#### Digestion and purification of pPICZαA and the esxA

The *esxA* and pPICZαA vector were digested with *Eco*RI and *Xba*I in a 50-µL solution consisting of 15 µL *esxA* sequence amplified by PCR or the pPICZαA vector, 1 µL *Eco*RI and *Xba*I (1 U), 5 µL buffer [10×M: 100 mM Tris-HCl (pH 7.5), 100 mM MgCl_2_, 10 mM dithiothreitol, and 500 mM NaCl], and 28 µL ddH_2_O. The samples were digested for 2 h at 37 °C, after which the digested products were stained and analyzed by 1 % AGE and purified using Esay Quik Gel Extraction Kit (Beijing TransGen Biotech. Co., Ltd, Beijing, China) according the operation manual.

#### Ligation of the esxA and the pPICZαA vector

The digested pPICZαA vector and *esxA* were ligated with T4 ligase in a 10-µL solution containing 2 µL pPICZαA (100 ng), 4 µL *esxA* (17 ng), 1 µL 10× ligase buffer, 0.2 µL T4 ligase (1 U), and 2.8 µL ddH_2_O. The ligation was completed during a 10-min incubation at 25 °C.

#### Subcloning of the pPICZαA-esxA recombinant plasmid and screening of positive clones

The pPICZαA-*esxA* recombinant plasmid was transinfected into competent *E. coli* Trans1-T1 cells, after which positive transformants were screened on solid LA medium containing bleomycin. A single positive colony was used to inoculate liquid LB medium containing bleomycin (1,000 µg/mL) in a centrifuge tube, which was then incubated for 1 h at 37 °C with shaking. The pPICZαA-*esxA* recombinant plasmid was extracted from the *E. coli* cells and sequenced to confirm its identity. The transformants containing pPICZαA-*esxA* were cultivated to amplified enough pPICZαA-*esxA* plasmids, after which the recombinant plasmid was purified using the Easy Pure Purification Kit (Beijing TransGen Biotech. Co., Ltd, Beijing, China), its quality were determined by 1 % agarose electrophoresis, and its concentration was determined on the ultra-micro ultraviolet spectrophotometer.

#### Linearization of pPICZαA-esxA

The pPICZαA-*EsxA* recombinant plasmid was linearized via a digestion with *Pma*I in a 50-µL solution comprising 2 µL *Pma*I (2 U), 5 µL CutSmart buffer, and 43 µL pPICZαA-*EsxA*. The digestion was completed during a 2.5-h incubation at 37 °C. The linearized product solution was concentrated to 15 µL and stored at − 20 °C.

#### Preparation of competent Pichia pastoris KM71H cells

*Pichia pastoris* KM71H cells (10 µL) stored in glycerol were recovered on ice and then used to inoculate 10 mL YPD medium in a 100 mL flask. The cells were cultured overnight at 37 °C with shaking (170 rpm). A 100-µL aliquot of the cells was used to inoculate 500 mL YPD medium in a 2 L flask. The cell culture was incubated overnight at 37 °C with shaking (170 rpm), after which the OD_595_ was 1.3–1.5. Competent cells were prepared as follows: (i) 50 mL KM71H cell suspension was centrifuged at 845×*g* for 5 min at 4 °C; (ii) After discarding the supernatant, the cells were resuspended in 50 mL sterile ddH_2_O pre-cooled on ice. The cell suspension was centrifuged again; (iii) After discarding the supernatant, 25 mL sterile water pre-cooled on ice was added to resuspend the cells, after which the cell suspension was centrifuged again; (iv) The supernatant was discarded and the cells were resuspended in 2 mL 1.0 M sorbitol pre-cooled on ice and then centrifuged again; (v) The cells were finally resuspended in 100 µL 1.0 M sorbitol pre-cooled on ice.

#### Transformation of competent *Pichia pastoris *cells with the linearized pPICZαA-esxA

Competent *P. pastoris* cells (80 µL) were added to an electroporation cuvette pre-cooled on ice, to which 100 µL linearized pPICZαA-*esxA* was added. The competent cells were electroporated for 4 ms at 1.5 kw, 25 µF, and 200 Ω, after which 1.0 mL 1.0 M sorbitol was added to the electroporation cup, which was incubated undisturbed for 2 h at 28 °C.

#### Screening of positive Pichia pastoris clones carrying pPICZαA-esxA

A 500 µL suspension of yeast cells transformed with linearized pPICZαA-*esxA* was spread on solid YPDS medium containing 500 µg/mL bleomycin. After a 5-day incubation at 28 °C, positive clones were selected for a PCR analysis.

#### Induction of esxA expression

A positive transformant was used to inoculate liquid BMGY medium, which was then incubated at 28 °C with shaking (250 rpm) until the OD_595_ reached 2–6 (16–18 h). The culture was centrifuged at 845×*g* at room temperature, after which the supernatant was discarded and the cells were resuspended in 1/10 volume of the original BMGY medium. The culture was then incubated as before. At 24-h intervals, 100 % methanol was added to the cell culture for a final concentration of 0.5 %. Cell samples were collected every 24 h after methanol was first added. Methanol was added four times and cell samples were collected four times. Each cell sample was centrifuged at 845×*g* for 5 min. The supernatant was transferred to a new centrifuge tube and stored at − 80 °C. After inducing *esxA* expression, to determine the optimal induction time, the abundance of the expressed protein in the supernatant was analyzed by sodium dodecyl sulfate polyacrylamide gel electrophoresis (SDS-PAGE) described previously (Yu et al. 2019b).

#### Identification of EsxA by mass spectrometry

The target band in the SDS-PAGE gel was excised and analyzed with the 5800 MALDI-TOF/TOF system (AB SCIEX) using the 384 Opti-TOF sample plate (123 mm × 81 mm) (AB SCIEX). The steps are summarized as follows:

Enzymatic hydrolysis: A sequencing-grade trypsin solution was added to the EP tube containing the excised SDS-PAGE gel strip. The enzymatic hydrolysis of the sample was completed during a 20-h incubation at 37 °C. Following the trypsin digestion, 100 µL 60 % acetonitrile (ACN)/0.1 % trifluoroacetic acid (TFA) was added to the tube, after which the sample was sonicated for 15 min and then lyophilized.

Mass spectrometry analysis: 1 µL dissolved sample was spotted on the sample plate. After the solvent dried, 0.6 µL supersaturated cinnamic acid matrix solution (50 % ACN/0.1 % TFA) was added to the corresponding target position and dried. The sample plate was treated with nitrogen gas and then placed in the sample injection target slot of the 5800 MALDI-TOF/TOF instrument (AB SCIEX). The Nd:YAG laser was used at a wavelength of 349 nm. The acceleration voltage was 2 kV. The positive ion mode and the automatic data acquisition mode were applied to collect data. The scanning range of the primary mass spectrum was 800–4,000 Da. A precursor ion with a signal-to-noise ratio greater than 50 was used for the secondary mass spectrum (MS/MS) analysis. Ten precursor ions were selected for each sample point, and the MS/MS spectra were derived from an accumulation of 2,500 laser shots. The collision energy was 2 kV, and the CID was turned off.

Protein database search: The original mass spectrometry data were used for database screening with the Mascot 2.2 software to identify proteins. The search parameters were as follows: (i) Database: *Paenibacillus* UniProt self-built library (strain NK3-4 predicted protein database); (ii) Search type: joint (MS + MS/MS); (iii) Enzyme: trypsin; (iv) Fixed correction: Carbamidomethyl (C); (v) Dynamic correction: Oxidation (M); (vi) Quantity: monoisotope; (vii) Protein quantity: unlimited; (viii) Peptide quality limit: ± 100 ppm; (ix) Fragment limit: ± 0.4 Da; (x) peptide charge: 1+; (xi) maximum missed detection: 1.

### Analysis of the induction of the plant hypersensitive response (HR) and reactive oxygen species (ROS) burst by EsxA

The HR and ROS test were conducted to detect the characteristic of EsxA consistent with those of elicitors (Borden and Higgins 2002; Mur 2007; Wei et al. 1992).

#### Induction of the HR

The expressed EsxA was purified by nickel column chromatography. Specifically, the protein was collected in approximately 30 mL eluant (50 mM Tris, 200 mM NaCl, 500 mM imidazole). After removing salt by dialysis method and concentrating by ultrafiltration, a 5-mL protein solution (2.0 mg/mL) was obtained. Tobacco, tomato, radish, and pakchoi were used as the test plants. Specifically, a previously described HR test was conducted(Yu et al. 2019b). Briefly, 20 µL EsxA solution (500 µg/mL) was injected into one side of each leaf, whereas bovine serum albumin (BSA) was injected into the other side as a control treatment. Additionally, another tobacco leaf injected with BSA served as a control for the ROS burst test. Analyses were repeated three times.

#### Induction of the ROS burst

At 24 h after the injections, the EsxA- and BSA-injected leaves and the untreated leaves growing above the treated leaves were collected and rinsed with ddH_2_O for the detection for ROS production. ROS were qualitatively detected by 3,3′-diaminobenzidine staining method (Frías et al. 2011). They were then immersed in a 3,3′-diaminobenzidine hydrochloride solution (1 mg/mL, pH 3.8). The samples were incubated undisturbed overnight in darkness, after which they were immersed in a solution comprising ethanol and glycerin [9:1 (v/v)] and incubated in a boiling water bath to decolorize the chlorophyll in the leaves. After the decolorization, 75 % glycerin was added to the leaf surface to flatten the leaves, which were then examined with a microscope. The detection of obvious brownish-red patches exclusively in the leaves of EsxA-injected plants (i.e., not in the control plants) indicated that EsxA induced the ROS burst.

### Determination of the effects of EsxA on rice seedling growth

The EsxA purified by nickel column chromatography was dissolved in PBS buffer. The seed-bud dipping method and the seedling dipping method were used to treat rice (*Oryza sativa* L. ssp. *japonica*) plants to determine the effects of EsxA on rice seedling growth.

#### Seed-bud dipping method

Coarse gravel was washed with water, then sterilized at 121 °C for 2 h, and the sterile wet gravelwas added to clean germination pots (10 cm diameter; 5 cm height), the gravel was 4.0 cm thick in every pots, total 6 pot were prepared.

Rice seeds was soaked in tap water at 25 °C for 3 days until the seeds fully absorbed water. During soaking the seeds, the water was renewed using fresh water once a day to prevent possible microbial contamination and provide sufficient oxygen for seed breath. After that, seeds were germinated at 30 °C for 24 h to make the germination neatly and consistently before sowing. Rice seed-buds [i.e., the bud (1 mm long) has broken through the seed coat] were uniformly sown on the surface of wet sterile gravel in 3 of the 6 pots (100 seed-buds per pot) that had been prepared. Then the seed-buds and the gravel were sprayed with EsxA solution [100 µg/mL in 50 mM PBS (pH 7.5)], to make the seed-busds fully, 0.5 mL of EsxA solution per pot. Then the seed-buds were covered with sterilized wet gravel (0.5 cm thick), which was then sprayed with the EsxA solution (0.5 mL per pot) to ensure the seed-buds keep being infiltrated with EsxA solution in gravel. Seed-buds treated with PBS instead of the EsxA solution that sowed in another 3 pots was as a control.

The germination pots were covered with clear Breathable lids and maintained at 28 °C for 24 h in an incubator in darkness. They were then transferred to a phytotron and incubated at 30 °C for 24 h, with a 12-h light cycle. After the incubation, ten seedlings were sampled randomly from each pot, and the shoot and root lengths of seedlings were determined. The analysis was completed with three replicates.

#### Seedling dipping method

Rice seeds were soaked and germinated in sterile water as described above, and placed in 6 germination pots (10 cm diameter; 5 cm height) lined with sterile filter paper, with each pot containing 100 germinated seeds. distilled water (10 mL) was poured into each pot, all pots then covered with breathable lids and maintained at 28 °C for 24 h in an incubator in darkness to ensure the length of seedling bud and root reach to 1 cm). The seedlings in 3 of the 6 pots were then sprayed with 1 mL EsxA solution (100 µg/mL). Control seedlings in the another 3 pots were sprayed with 50 mM PBS (pH 7.5). The pots were incubated at room temperature (day: 25 °C; night: 18 °C) for 48 h, with a 12-h light cycle. After the incubation, ten seedlings were sampled randomly from each pot, and the shoot and root lengths were determined. The analysis was completed with three replicates.

### Data analysis

SPSS 13.0 software (Chicago, USA) was used to perform a one-way ANOVA to assess the significance of the differences between two group of variables, the significant difference was determine at 0.05 levels. Paired-samples T test was conducted to detect whether the data violate the assumption. Bar graphs were prepared using Excel 2010.

## Results

### Cloning and expression of the esxA and the functional characterization of the encoded protein

#### Genomic DNA extraction and PCR amplification of the esxA

**Fig. 2:**
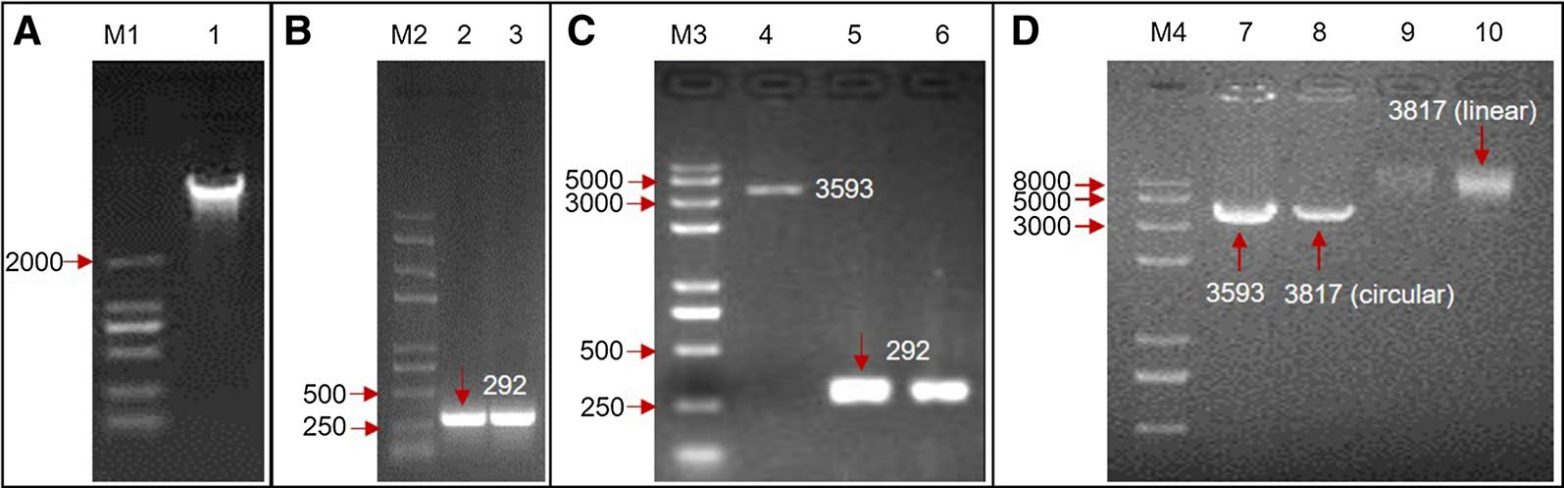
Agarose gel electrophoretic analysis of DNA. In panel **A** *Paenibacillus terrae* NK3-4 genomic DNA; in panel **B** *esxA* amplification; in panel **C** purified vector (lane 4) and *esxA* (lane 5 and 6) preparation; in panel **D** construction and linearization of pPICZαA-*esxA.* M: marker (M1: DL2000; M2, M3 and M4: D8000); lane 1: genomic DNA; lane 2, 3: amplified *esxA*; lane 4: pPICZαA vector; lane 5, 6: amplified *esxA* product recovered from the agarose gel; lane 7: circular pPICZαA; lane 8: circular pPICZαA-*esxA*; lane 9, 10: linear pPICZαA-*esxA*; DNA lanes labled with molaculor weight (bp)

*Paenibacillus terrae* strain NK3-4 genomic DNA was analyzed by agarose gel electrophoresis and spectrophotometrically. The genomic DNA bands were clear and there were no obvious signs of degradation (Fig. 2A). The OD_260_/OD_280_ ratio was between 1.8 and 2.0. The genomic DNA was free of proteins and RNA. These results confirmed the quality of the genomic DNA was appropriate for the subsequent PCR amplification. The analysis of the amplified product by agarose gel electrophoresis revealed a fragment slightly longer than 250 bp was cloned from the genomic DNA, which was consistent with the size of the *esxA* (Fig. 2B). Thus, the EsxA sequence was successfully amplified by PCR. After purifying the amplified fragment from the gel, it was once again analyzed by agarose gel electrophoresis. The corresponding band was bright and the DNA concentration was sufficiently high. Additionally, the pPICZαA vector was successfully amplied and purified (Fig. 2C). The amplified *esxA* sequence and the pPICZαA vector were suitable for the following experi ments.

#### Construction and linearization of the pPICZαA-esxA recombinant plasmid

The amplified *esxA* and pPICZαA digested with *Eco*RI and *Xba*I were ligated. The agarose gel electrophoresis analysis of the ligation product indicated that the empty vector (pPICZαA) and the pPICZαA-*EsxA* recombinant plasmid comprised 3,000–5,000 bp, which was consistent with the predicted sizes [3593 (pPICZαA) – 61 (length of restriction sites between *Eco*RI and *Xba*I) + 285 (length of the *Eco*RI and *Xba*I digested PCR product showed in Fig. 1) = 3817 bp]. Moreover, the empty vector migrated slightly faster than pPICZαA*-esxA*, implying the recombinant plasmid was successfully constructed (Fig. 2D, lane 7 and 8). After pPICZαA-*esxA* was digested, it migrated through the agarose gel significantly more slowly than the circular recombinant plasmid, indicating that pPICZαA-*esxA* had been successfully linearized (Fig. 2D, lane 9 and 10).

#### Transformation of yeast cells with pPICZαA-esxA and analysis of the induced EsxA expression

**Fig. 3:**
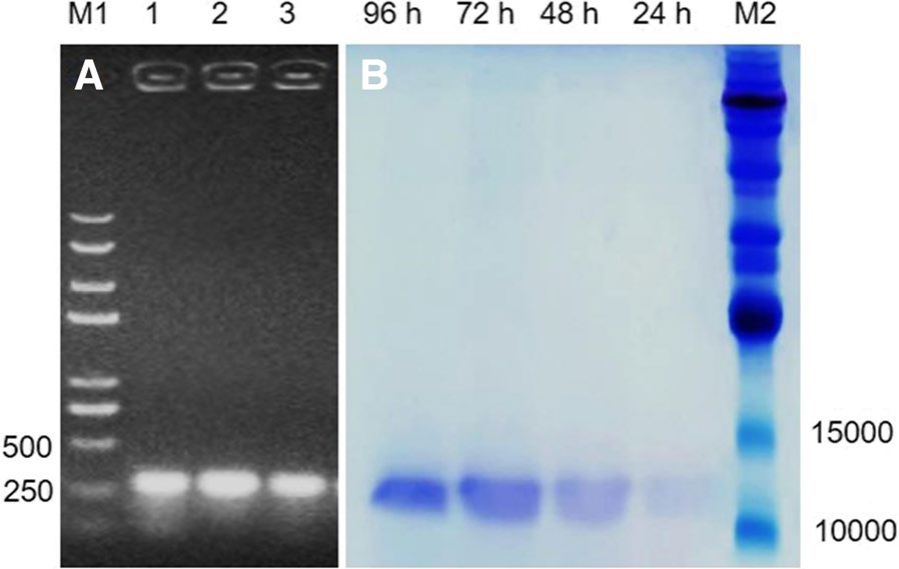
Detection of transformants by PCR (**A**) and an analysis of expressed proteins by SDS-PAGE (**B**). In panel **A** M1: DNA marker (D8000), 1 − 3: PCR analysis of transformants carrying the *esxA*; in panel **B** 24, 48, 72, and 96 h: induction periods; M2: protein marker (P0060). DNA (bp) and ptoerin lanes (Da) labelled with molecular weight

After examining transformants by colony PCR using *esxA*-specific primers, an agarose gel electrophoresis analysis revealed an amplified product that was longer than 250 bp, indicating the cells were correctly transformed with pPICZαA-*EsxA* (Fig. 3A). The confirmed transformants were cultured and induced for 24, 48, 72, and 96 h. An SDS-PAGE analysis indicated that specific proteins were expressed during different induction periods, which was a single (wide) band below 15 kDa and above 10 kDa, was consistent with the predicted sizes [10276.53 (EsxA) + 930.96 (6His-tag) = 11207.49 Da]. The protein expression levels were significantly higher at 72 and 96 h than at 24 and 48 h (Fig. 3B).

#### Identification of EsxA by mass spectrometry

The band in the SDS-PAGE gel with the expected size for EsxA was excised from the gel and analyzed by mass spectrometry, which confirmed the expressed protein was EsxA. Most of the amino acids in this protein have been identified. The five detected peptide fragments were ILITPEQVDQVANQF, EQSQQIVSSLTQSIS, GMEGQWEGMTKQR, QRFFQEFQEASK, and TLNSISQELTAI. The MS/MS spectra of two peptides and the distribution of these peptides in EsxA are presented in Figure. The amino acid sequence included the conserved WXG motif Trp44(W) − Glu45(E) − Gly46(G).

**Fig. 4:**
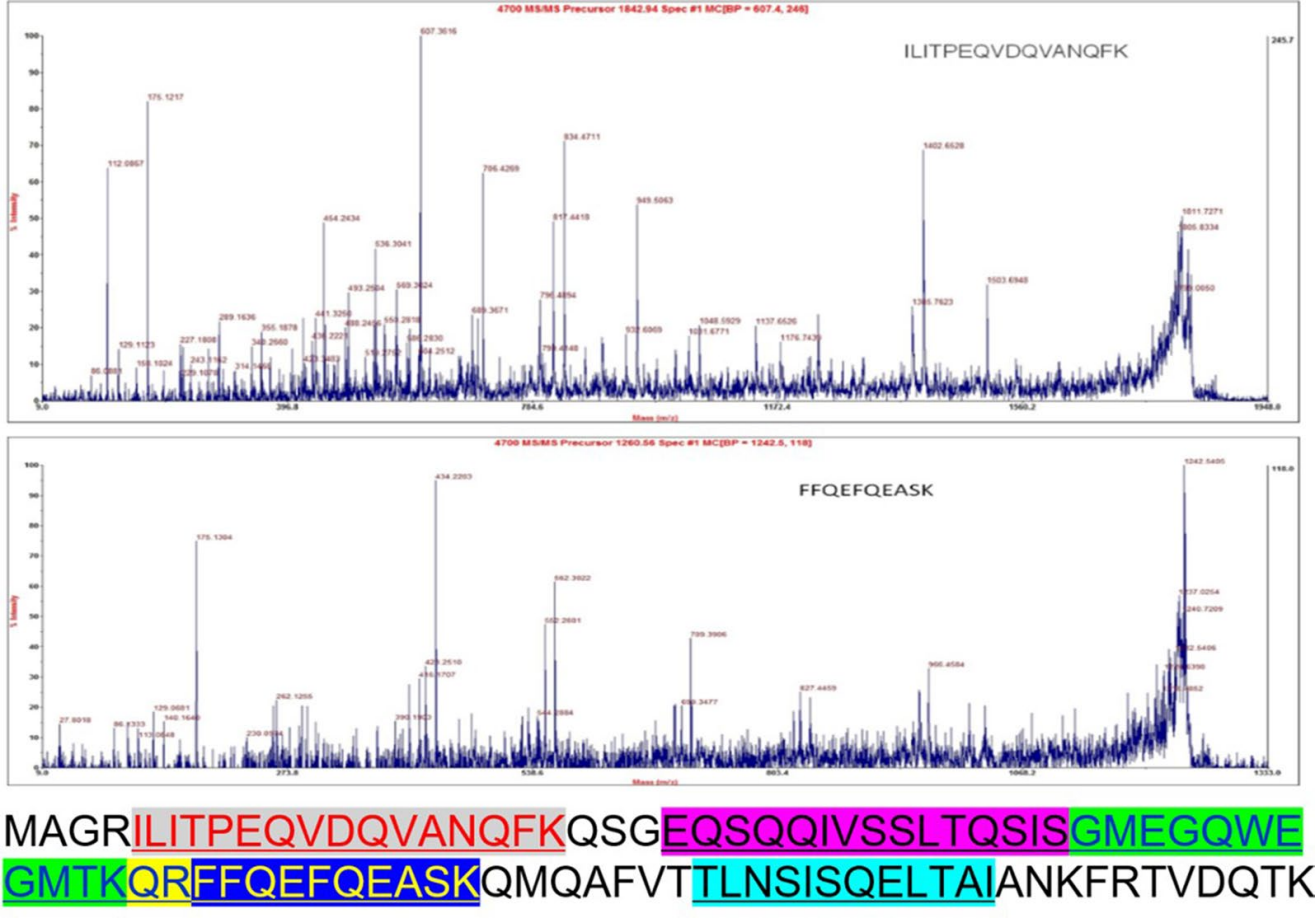
Secondary mass spectra of EsxA peptides as well as the identified peptide fragments and their positions. The underlined amino acids indicate the identified sequences. The different font colors and different backgrounds represent the identified peptide fragments

#### Induction of the plant HR and ROS burst by EsxA

**Fig. 5:**
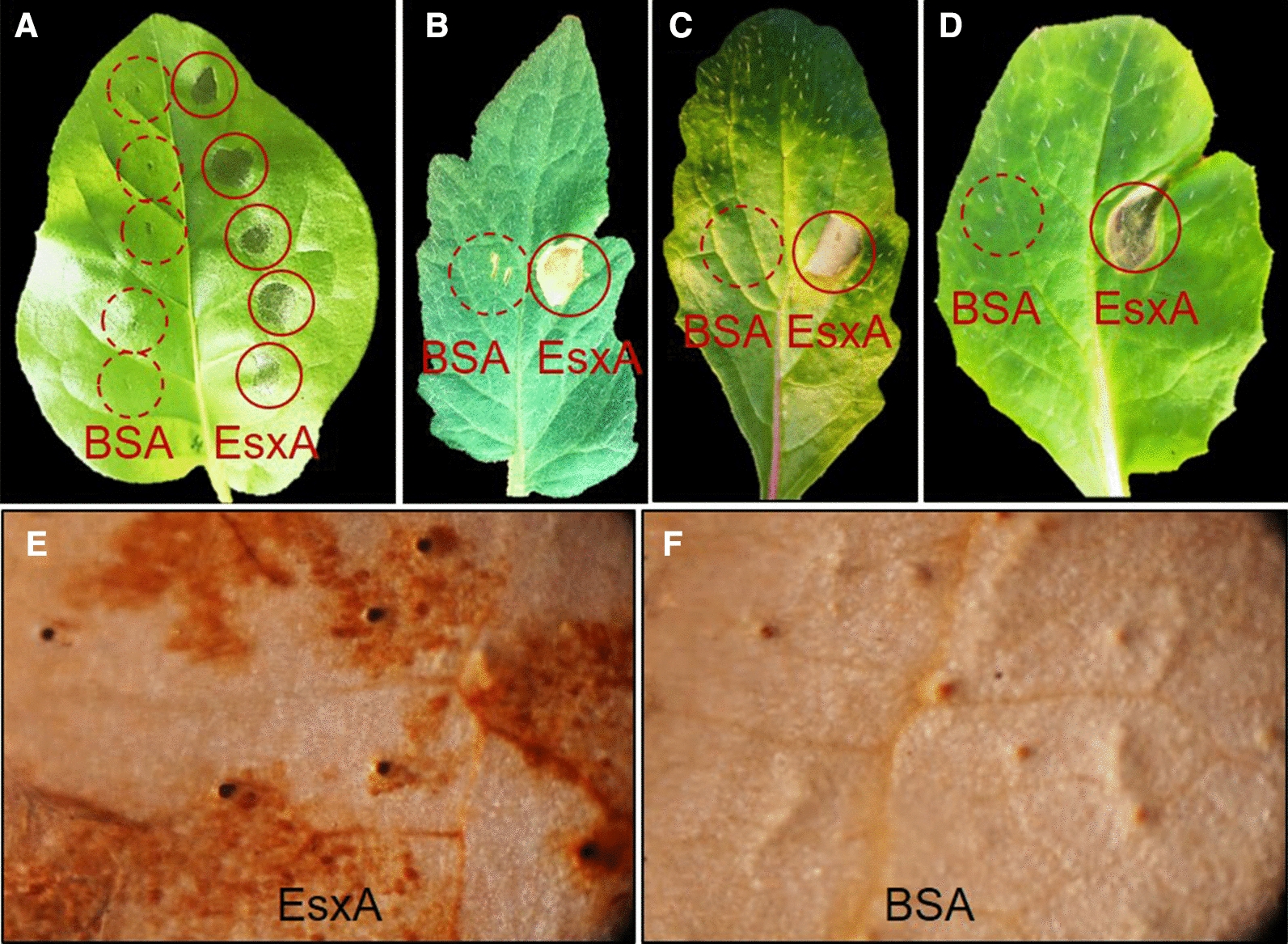
Hypersensitive reaction in various plants and reactive oxygen species burst in tobacco leaves treated with EsxA. **A**–**D** hypersensitive response in leaves of *Nicotiana tabacum*, *Solanum lycopersicum*, *Scrophularia ningpoensis*, and *Brassica rapa* L. ssp. *chinensis* injected with EsxA, respectively. Con represents control (BSA), EsxA represents EsxA injected; **E**, **F** Detection of reactive oxygen species in the tobacco leaves growing above those injected with EsxA and in control leaves injected with bovine serum albumin, respectively

An analysis of the tobacco, tomato, radish, and pakchoi plants revealed that EsxA can induce the HR in these species (Fig. 4A–D). Moreover, in addition to the injected leaves, EsxA also induced the ROS burst in untreated leaves growing above the injected leaves (Fig. 5E). In contrast, the ROS burst was not detected in the BSA-injected control leaves (Fig. 5F). These results suggest that EsxA has characteristics consistent with those of an elicitor.

#### Effects of EsxA on rice seedling growth

**Fig. 6:**
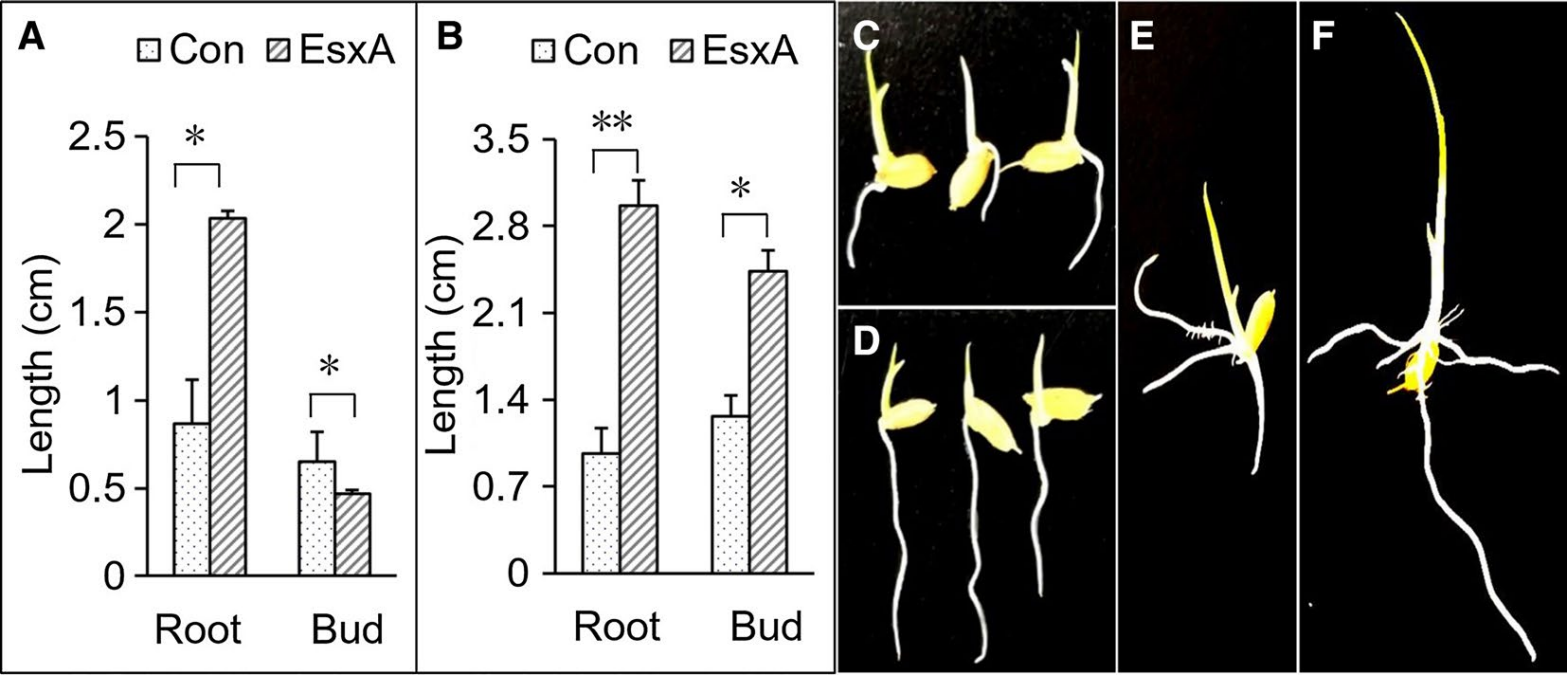
Effects of EsxA on rice seedling growth. **A**: seed-bud dipping experiment; **B**: seedling dipping experiment; **C,** **D**: control and EsxA treatments in the seed-bud dipping experiment, respectively; **E, F**: control and EsxA treatments in the seedling dipping experiment. * and ** represents differences are significantly at the levels of P = 0.01 and P = 0.001, respectively

In the seed-bud dipping experiment, EsxA (100 µg/mL) significantly affected rice seedling growth, with the treatment increasing the root length by 1.35-times (F = 29.878, P = 0.005) and decreasing the shoot length by 28.8 % (F = 30.250, P = 0.005) (Fig. 6A, C, D). In the seedling dipping experiment, the EsxA treatment promoted seedling growth. Specifically, the EsxA-treated roots and shoots were respectively 2.6-times (F = 112.500, P < 0.001) and 1.7-times (F = 38.281, P = 0.003) longer than the control roots and shoots (Fig. 6B, E, F). These observations confirmed that EsxA can promote the growth of rice plants, especially the roots.

## Discussion

In this study, the *esxA* of *P. terrae* strain NK3-4 was cloned. Additionally, the EsxA protein was produced in a yeast expression system and its rice growth-promoting effects were confirmed for the first time. Previous research on the *esxA* did not involve a functional characterization in *Paenibacillus* strains. Thus, its potential role in the mechanism underlying the plant growth-promoting activities of *Paenibacillus* strains remained unknown. Earlier investigations on EsxA focused on its effects on the pathogenicity of animal pathogens or its induction of immune responses in disease-resistant animals (Ma et al. 2015; Zhou et al. 2013). The results of the current study imply that the mechanism regulating the plant growth-promoting effects of *Paenibacillus* strains, including NK3-4, may involve the secretion of EsxA. Accordingly, EsxA may be useful as a new protein elicitor that can regulate plant growth. Recent studies proved that protein elicitors can stimulate plant metabolism to modulate plant growth (Darwati et al. 2018; Shen et al. 2019). Moreover, interacting proteins that directly or indirectly bind to elicitors have been identified in plants (Mario et al. 2017). This interaction induces a series of downstream signal transduction pathways to stimulate secondary metabolic activities related to plant growth regulation (Zhao et al. 2005; Liu et al. 2018; Tang et al. 2014; Cui et al. 2017). For example, the application of a yeast elicitor reportedly enhances the production of phenolic acids and tanshinones in *Salvia miltiorrhiza*, while also increasing hairy root growth (Chen et al. 2001). The mechanisms by which elicitors promote plant growth are complex. In response to an elicitor, plant growth and metabolism may be affected via the cross-talk among different signaling pathways (e.g., cross-talk between the elicitor and jasmonate and between jasmonate and ethylene signaling pathways) and the interactions between these pathways and ROS as well as the integration of multiple signaling pathways and transcription factors (Cheng et al. 2018). These signaling components are connected in an elicitor signaling network and transduce elicitor signals at the transcriptional and metabolic levels, thereby influencing plant secondary metabolism to further regulate plant growth (Zhao et al. 2005; Cheplick et al. 2018).

In this study, we determined that EsxA likely contributes to the plant growth-promoting activities of specific rhizobacteria, implying EsxA may be useful as a plant growth regulator. The fact that EsxA can promote plant growth suggests certain molecular interactions occur between EsxA and plants. Future research should focus on these interactions to elucidate the EsxA-associated plant growth-promoting mechanism and possibly provide the theoretical basis for the commercial application of EsxA as a growth regulator.

## Data Availability

Please contact the authors for all requests.

## Abbreviations

ACN: Acetonitrile
AGE: Agarose gel electrophoresis
BSA: Bovine serum albumin
HR: Hypersensitive response
ROS: Reactive oxygen species
TFA: Trifluoroacetic acid
